# Abnormalities of white matter network properties in middle-aged and elderly patients with functional constipation

**DOI:** 10.3389/fneur.2024.1357274

**Published:** 2024-03-27

**Authors:** Hou Xueyan, Ai Qi, Song Chunming, Zhi Yu, Weng Wencai

**Affiliations:** ^1^Department of Radiology, Xinhua Hospital Affiliated to Dalian University, Dalian, Liaoning, China; ^2^Department of Radiology, The First Affiliated Hospital of Dalian Medical University, Dalian, Liaoning, China; ^3^Graduated School, Tianjin Medical University, Tianjin, China; ^4^Pelvic Floor Center, Xinhua Hospital Affiliated to Dalian University, Dalian, Liaoning, China

**Keywords:** functional constipation, middle aged and elderly people, graph theory, white matter network, topological properties

## Abstract

**Purpose:**

To explore white matter network topological properties changes in middle-aged and elderly patients with functional constipation (Functional Constipation, FC) by diffusion tensor imaging (DTI), and to evaluate the correlation between the abnormal changes and clinical data.

**Methods:**

29 FC patients and 31 age- and sex-matched healthy controls (HC) were recruited. Magnetic resonance imaging and clinical data were collected. The white matter network changes in FC patients were analyzed using deterministic fiber tracking methods, graph theory algorithms, and partial correlation analysis with clinical data.

**Results:**

The nodal clustering coefficient and nodal local efficiency of FC patients in the right orbital inferior frontal gyrus, right medial superior frontal gyrus, right rectus muscle, right hippocampus, left paracentral lobule and left temporal pole, and the nodal clustering coefficient in right orbital superior frontal gyrus, left cuneus lobe and right superior occipital gyrus, the nodal local efficiency in the right medial and paracingulate gyrus, right precuneus and right dorsolateral superior frontal gyrus of FC patients are lower than that of HC. The nodal local efficiency and clustering coefficient of FC patients in left hippocampus, left amygdala, right parietal inferior limbic angular gyrus and right angular gyrus, the nodal local efficiency in the right fusiform gyrus, left supplementary motor cortex and the nodal efficiency in the left lateral temporal gyrus and right orbital middle frontal gyrus (ORBmid.R) of FC patients are higher than that of HC. The nodal efficiency of ORBmid.R in FC was positively correlated with the Patient Assessment of Constipation quality of life questionnaire (PAC-QoL).

**Conclusion:**

Middle-aged and elderly FC patients have differences in the nodal level properties in the limbic system, supplementary motor cortex, and default mode network brain regions, and the nodal efficiency of ORBmid.R was positively correlated with the PAC-QoL score, revealing that FC may be related to the abnormal processing of visceral sensorimotor in ORBmid.R and providing potential imaging diagnostic markers and therapeutic targets for middle-aged and elderly FC patients.

## 1 Introduction

Functional constipation (FC) is a common functional gastrointestinal disease (1); it is characterized by infrequent defecation, straining, lumpy or hard stools, and sensation of incomplete defecation, often accompanied by abdominal distension and abdominal pain (2). Long-term constipation not only increases the prevalence of hemorrhoids and colorectal cancer (3), but also causes diseases related to the central nervous system (such as cerebrovascular diseases, anxiety, depression, and so on.) (4), which in turn leads to changes in structure and function. It has severely impacted the individual’s mental state, quality of life, and even social health and economy. Therefore, it is necessary to study the potential neuroimaging mechanisms of FC.

The current worldwide prevalence of FC is approximately 0.7–79% (median 16%) (5). According to the meta-analysis report on the prevalence of FC in 2017, the total prevalence of FC in China was 8.5%. The overall prevalence of FC showed a steady upward trend between 1991 and 2020, reaching a maximum of 10.9% (6). In addition, with increasing age, the secretory function of human digestive glands continues to degenerate, the sensitivity of the buttocks muscles continues to decrease, the colorectal muscle layer gradually becomes thinner, and the muscle tension of the intestinal smooth muscles also continues to decrease. Therefore, middle-aged and older adults can easily cause constipation, and the overall prevalence of FC increases with age (5). Therefore, China, as a country with an aging population, focusing on functional constipation in middle-aged and older adults is crucial in improving their quality of life.

FC is a non-organic disease, and the research on its etiology and pathogenesis still needs to be more thorough in functional gastrointestinal disorders. However, its pathogenesis may be related to visceral hyposensitivity, abnormal gastrointestinal motility, genes, and brain-gut axis interaction (7), the most important of which is the gut-brain axis (GBA) theory. GBA refers to the brain and gastrointestinal bidirectional pathway, brain and gut are interconnected by immune, nerve, endocrine and microbial metabolism. However, the specific interaction mechanism between central functional activity and intestinal function in FC patients remains uncertain. Neuroimaging is a vital tool to non-invasively observe and detect the subtle structure and functional activities of the brain using multimodal imaging techniques (such as magnetic resonance, electroencephalography, and magnetoencephalography), which can essentially reveal the potential relationship between changes in brain structure and function and diseases, providing an essential basis for in-depth exploration of the neuroimaging mechanism of FC (8).

Neuroimaging has used multimodal imaging methods and data analysis methods to confirm the structural and functional abnormalities (9–13) in somatic and visceral perception, emotional arousal, default mode network, salient network, and sensorimotor network related brain regions in patients with functional gastrointestinal diseases. Liu et al. used RS-fMRI combined with graph theory analysis to show reduced nodal properties in the thalamus, ACC and supplementary motor cortex involved in somatic, emotional processing and motor control brain regions, and speculated that FC is associated with abnormal connectivity in the thalamo-cortical network (14). In addition, Pei and other scholars using DTI, deterministic fiber tracking method and combined with graph theory analysis found increased nodal properties in the left superior frontal orbital, the right middle frontal orbital, the right anterior cingulate and cingulate, and decreased nodal properties in the left caudate nucleus and left thalamus in FC patients, and the duration of FC was negatively correlated with nodal betweenness in the left thalamus. This study reveals that abnormal visceral sensory processing in FC is associated with abnormal changes in the topological properties of the thalamic white matter network from the perspective of human connectomics (15). However, whether FC in middle-aged and elderly patients has abnormal white matter structure among these brain regions remains unclear.

## 2 Materials and methods

### 2.1 FC patients

In this study, 29 FC patients were recruited from the Pelvic Floor Clinic, Xinhua Hospital Affiliated to Dalian University, from March 20, 2021, to October 31, 2022. FC is diagnosed by gastroenterologists experienced in the diagnosis of functional gastrointestinal disorders using Rome IV criteria (2).

The entry criteria are as follows:

(1) Meeting the diagnostic criteria of Rome IV functional constipation;
(2) Aged 45–79 years old, right-handed;
(3) Not taking gastrointestinal prokinetic agents or laxatives at least 15 days before enrollment;
(4) Not participating in other clinical trials in the past 3 months.

The exclusion criteria are as follows:

(1) Organic diseases such as the digestive system and hematopoietic system are found in the routine examination;
(2) History of gastrointestinal surgery or head trauma with loss of consciousness;
(3) Have a history of severe mental disorders, such as anxiety and depression;
(4) Those with a history of dysmenorrhea;
(5) Contraindications to MR scanning;
(6) Refusal to sign the informed consent form.

### 2.2 HC

Thirty one sex- and age-matched healthy subjects were recruited for this study from March 10, 2022, to October 31, 2022.

The entry criteria are as follows:

(1) Aged 45–79 years old, right-handed;
(2) No organic or functional disease was found in the routine examination;
(3) Have not participated in other clinical trials in the past 3 months.

The exclusion criteria are as follows:

(1) Any discomfort in the digestive system;
(2) have a history of dysmenorrhea;
(3) History of gastrointestinal surgery or head trauma with loss of consciousness;
(4) Contraindications to MR scanning;
(5) Refusal to sign the informed consent.

### 2.3 Clinical evaluation

The disease severity was independently assessed by two experienced clinicians at least using the Patient with Constipation Clinical Rating Scale and the Patient with Constipation Quality of Life Questionnaire (PAC-QoL). Since mental states such as anxiety/depression may affect changes in brain function and structure, this study used the Hamilton Anxiety Scale (HAMA) and the Hamilton Depression Scale (HAMD) to assess the anxiety and depression-related symptoms of the subjects.

### 2.4 MRI data acquisition

All subjects underwent MRI scanning on a 1.5 T MRI scanner (Siemens 1.5 T MR scanner) using a 12-channel phased array head coil at the Department of Radiology, Xinhua Hospital Affiliated to Dalian University. During scanning, subjects were in the supine position, wearing non-magnetic earphones and using a soft cushion to stabilize the head.

The scanning parameters are as follows: (1) DTI scanning parameters: Spin-echo sequence is used, diffusion-sensitive gradients are applied in 20 directions, including a diffusion-weighted imaging (b = 0) without gradient direction and 19 along-diffusion sensitivity coefficients: 1,000s/mm (2) gradient direction imaging, pulse repetition time (RepetitionTime, TR) = 6,700 m/s, echo time (Echo Time, TE) = 98.1 m/s, slice thickness = 3 mm, a total of 45 layers, field of view (field of view, FOV) = 256 × 256 mm (2), flip angle (flip angle, FA) = 90°, matrix (matrix) = 128 × 128. (2) MRI scan parameters: T1-weighted sagittal scan images were obtained using fast echo sequence, TR = 1,940 m/s, TE = 3.3 m/s, FOV = 256 × 256 mm (2), slice thickness = 3 mm, slice distance =0 mm, FA = 15°, matrix = 256 × 192.

### 2.5 MRI data preprocessing and network construction

#### 2.5.1 MRI data preprocessing

Use the PANDA software installed on the Linux system and MATLAB to preprocess the diffusion-weighted imaging data. The specific preprocessing operation process is as follows: convert the DICOM image format to the NIFTI format; head motion/eddy current correction; gradient direction correction; obtain the brain mask: go except for brain tissue, improving the accuracy of spatial registration, limiting the scope of analysis, and reducing the amount of calculation; and use DTIFIT to calculate fractional anisotropy (FA).

#### 2.5.2 Network construction

Brain networks can be constructed by two essential elements: nodes and edges. Nodes of structural brain networks represent brain regions, and network edges represent morphological connections. Specifically, we use the network node definition function of PANDA software, and the human brain prior map Automated Anatomical Labeling90 (ALL90) to divide the brain into 90 regions of interest (ROI) and use an inverse algorithm from T1 to standard space to register AAL 90 to DTI. Furthermore, through the deterministic fiber tracking technology based on the Fiber Assignment by Continuous Tracking (FACT) algorithm [if the angle >45° or FA < 0.2 (15), the tracking is terminated] to determine the anatomical connection between network nodes, and calculate the weighted connection matrix based on the number of fiber connections (Fiber Number, FN).

### 2.6 Network analysis

Graph theory is one of the main tools for studying the topological organization of brain networks. We use the Network Analysis function module in the graph theory network analysis toolbox GRETNA to calculate the topological properties of brain networks. The FN threshold is set to 3 (16), and the global properties include Small-world parameters for clustering coefficient and characteristic path length, local and global efficiency, modularity, coordination, synchronization, and hierarchy. Nodal properties include nodal degree, nodal efficiency, nodal cluster coefficient, etc.

### 2.7 Statistical analysis

#### 2.7.1 Statistical analysis of network topological properties

Using the function of matrix comparison in GRETNA, the two-sample *t*-test was used to compare the differences in white matter network topology between FC patients and HC groups. Multiple comparisons were corrected using the Bonferroni method, and statistical significance was assigned at 0.05.

#### 2.7.2 Clinical data analysis

SPSS version 26.0 was used for the statistical analysis of clinical variable data. For measurement data (age, education, HAMA and HAMD), data conforming to a normal distribution were compared between groups using a two-tailed independent sample *t*-test. For count data (sex), differences between groups were compared using the chi-square test. Statistical significance was assigned at 0.05.

#### 2.7.3 Correlation analysis

Partial correlation analyses between neuroimaging indicators and disease severity (duration, clinical rating scale, and PAC-QoL) were performed with age, sex, HAMA, and HAMD as covariates. Nodal efficiency is more sensitive and effectively reflects the transfer of information flow in the network, so it was chosen as a representative indicator to examine the correlation between network properties and clinical variables.

### 2.8 Ethical approval

Ethical approval for all patients in our research was given by the Ethics Committee of Affiliated Xinhua Hospital of Dalian University (2021–085-01).

## 3 Results

### 3.1 Demographic and clinical data

The comparison results of demographic characteristics (age, sex, and years of education), clinical variables (duration, clinical rating scale, and PAC-QoL) and psychiatric indicators (HAMA and HAMD) between the FC and HC groups are as follows (Table 1). For age, sex and years of education, there was no significant difference between the FC and HC groups (*p* > 0.05). The HAMA and HAMD scores of FC patients were significantly higher than those of HC (*p* < 0.05).

**Table 1:** Demographic and clinical characteristics of FC and HC groups.

	FC(*n* = 29) Mean ± SD	HC(*n* = 31) Mean ± SD	*p*-value
Age (years)	66.66 ± 8.56	64.06 ± 9.02	0.259^a^
Gender (M/F)	10/19	10/21	0.885^b^
Education (years)	11.45 ± 2.66	11.71 ± 3.22	0.604^a^
HAMA	8.24 ± 5.19	2.16 ± 2.60	0.000^*a^
HAMD	7.76 ± 5.82	1.39 ± 1.05	0.000^*a^
Duration (months)	4.00(9.00)	/	
Clinical scoring scale	12.00(6.00)	/	
PAC-Qol	64.79 ± 19.25	/	

*P < 0.05. ^a^Indicates the p-value was obtained by two-tailed independent-sample *t*-tests. ^b^Indicates the *p*-value was obtained by chi-square test.

### 3.2 Global topological properties of white matter network

**Figure 1:**
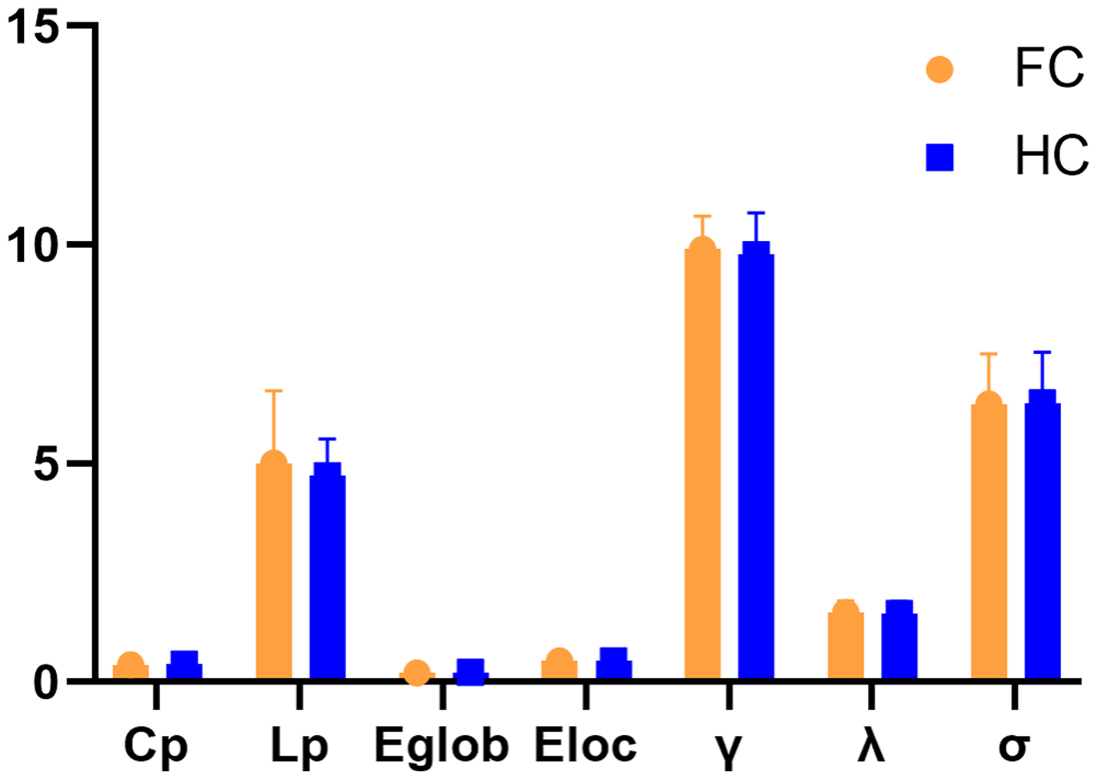
Differences in global topological characteristics of the white matter network between the FC and HC groups.

Within the defined thresholds, both groups exhibit small-world network architectures (γ > > 1, λ ≈ 1, and = γ/λ > 1) in WM networks. However, no statistically significant differences were seen for these global topological features Cp, Lp, Eglob, Eloc, λ, γ, and σ (Figure 1).

### 3.3 Nodal topological properties of white matter network

**Figure 2:**
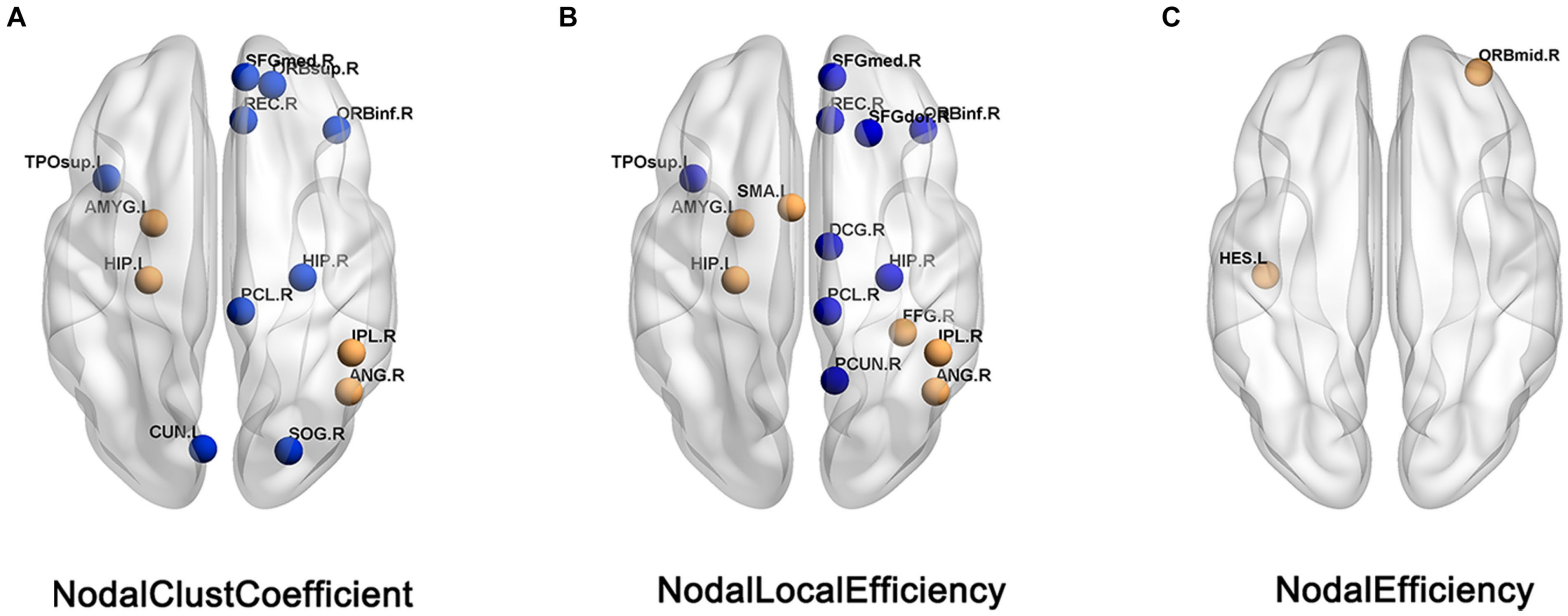
Brain regions showed difference in nodal topological characteristics between the FC and HC groups. **(A)** Nodal clust coefficient. **(B)** Nodal local efficiency. **(C)** Nodal efficiency, the blue nodes indicates the value of FC< the value of HC, the orange nodes indicats the value of FC > the value of HC.

Compared with HC patients, the nodal clustering coefficient and nodal-local efficiency of FC patients in the right orbital inferior frontal gyrus (ORBinf.R), right medial superior frontal gyrus (SFGmed.R), right Rectus muscle (REC.R), right medial and right hippocampus (HIP.R), left paracentral lobule (PCL.L) and left temporal pole (TPOsup.L) are significantly decreased (*p* > 0.05); The nodal clustering coefficient in the right orbital superior frontal gyrus (ORBsup.R), left cuneus lobe (CUN.L) and right superior occipital gyrus (SOG.R), the nodal local efficiency in the paracingulate gyrus (DCG.R), right precuneus (PCUN.R) and right dorsolateral superior frontal gyrus (SFGdor.R) of FC patients are significantly decreased (*p* > 0.05); The nodal local efficiency and clustering coefficient of FC patients in left hippocampus (HIP.L), left amygdala (AMYG.L), right parietal inferior limbic angular gyrus (IPL.R) and right angular gyrus (ANG.R) are significantly increased (*p* > 0.05); The nodal local efficiency in the right fusiform gyrus (FFG.R), left supplementary motor cortex (SMA.L) and the nodal efficiency in the left lateral temporal gyrus (HES.L) and right orbital middle frontal gyrus (ORBmid.R) of FC patients are significantly increased (Table 2; Figure 2).

**Table 2:** Differences in nodal topological characteristics of the white matter network between the FC and HC groups.

Brain regions	Category	NodalClustCoefficient			NodalLocalEfficiency			NodalEfficiency		
		FC	HC	P	FC	HC	P	FC	HC	P
FC<HC										
SFGdor.R	Association				0.36 ± 0.15	0.44 ± 0.16	0.0394			
ORBsup.R	Paralimbic	0.35 ± 0.28	0.55 ± 0.39	0.0021						
ORBinf.R	Paralimbic	0.38 ± 0.23	0.51 ± 0.24	0.0237	0.46 ± 0.25	0.59 ± 0.25	0.0218			
SFGmed.R	Association	0.00 ± 0.00	0.28 ± 0.42	0.0001	0.00 ± 0.00	0.30 ± 0.45	0.0001			
REC.R	Paralimbic	0.34 ± 0.28	0.58 ± 0.39	0.0004	0.40 ± 0.32	0.62 ± 0.40	0.0022			
DCG.R	Paralimbic				0.37 ± 0.12	0.44 ± 0.11	0.0185			
HIP.R	Limbic	0.18 ± 0.06	0.23 ± 0.07	0.0040	0.23 ± 0.10	0.32 ± 0.12	0.0009			
CUN.L	Association	0.71 ± 0.34	0.88 ± 0.25	0.0499						
SOG.R	Association	0.33 ± 0.07	0.38 ± 0.10	0.0275						
PCUN.R	Association				0.21 ± 0.09	0.26 ± 0.09	0.0450			
PCL.L	Association	0.63 ± 0.25	0.73 ± 0.20	0.0004	0.76 ± 0.28	0.85 ± 0.17	0.0004			
TPOsup.L	Paralimbic	0.46 ± 0.18	0.60 ± 0.15	0.0031	0.53 ± 0.21	0.72 ± 0.18	0.0007			
FC>HC										
ORBmid.R	Paralimbic							0.17 ± 0.03	0.12 ± 0.08	0.0055
SMA.L	Association				0.44 ± 0.15	0.37 ± 0.10	0.0228			
HIP.L	Limbic	0.17 ± 0.05	0.14 ± 0.04	0.0046	0.23 ± 0.07	0.18 ± 0.07	0.0043			
AMYG.L	Subcortical	0.84 ± 0.37	0.52 ± 0.51	0.0101	0.84 ± 0.37	0.52 ± 0.51	0.0101			
FFG.R	Association				0.41 ± 0.12	0.35 ± 0.11	0.0280			
IPL.R	Association	0.41 ± 0.14	0.34 ± 0.11	0.0247	0.56 ± 0.24	0.43 ± 0.19	0.0128			
ANG.R	Association	0.36 ± 0.13	0.21 ± 0.19	0.0000	0.53 ± 0.22	0.29 ± 0.29	0.0000			
HES.L	Primary							0.11 ± 0.09	0.05 ± 0.08	0.0110

### 3.4 Partial correlation between topological properties of abnormal white matter network and clinical variables

**Figure 3:**
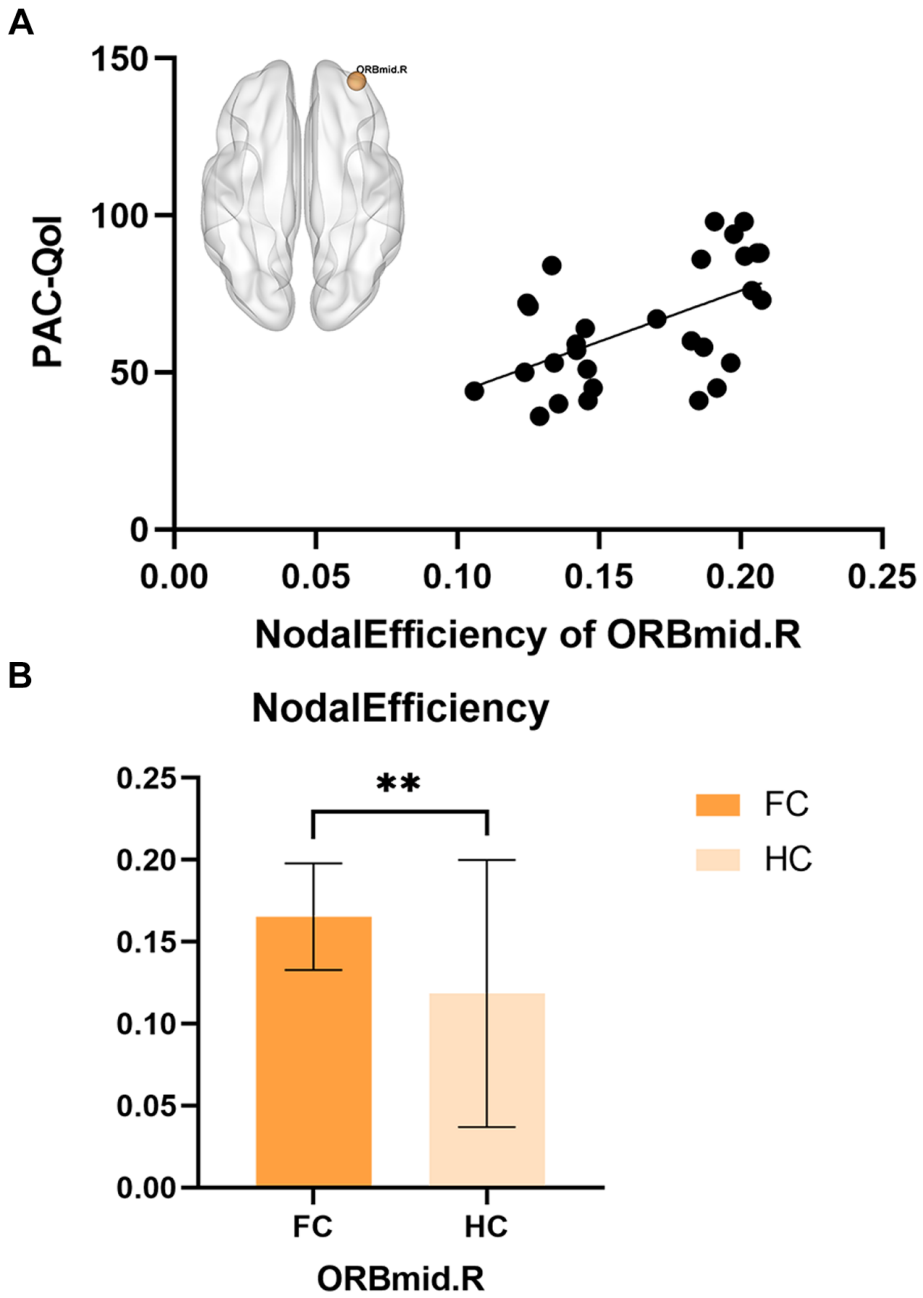
**(A)** The correlations between nodal efficiency of ORBmid.L in FC and PAC-QoL. **(B)** The differences in nodal efficiency of ORBmid.L between FC and HC, **p < 0.01.

Partial correlation analysis showed that in the FC patient group, the nodal efficiency of ORBmid.R was positively correlated with the PAC-QoL score (*r* = 0.55, *p* = 0.002) (Figure 3).

## 4 Discussion

This study is the first to explore abnormalities in topological properties of white matter networks in middle-aged and elderly FC patients. The results showed that middle-aged and elderly patients with FC and HC had significant differences in the nodal topological properties in the limbic system, supplementary motor cortex and default mode network brain regions. No significant differences in global properties were found.

### 4.1 Global topological properties of FC patients

The human brain is a complex network composed of many brain regions and white matter fibers. White matter fibers not only account for a large proportion of brain structure but also play an important role in the process of information transmission between brain regions. Topological properties of white matter networks visualize how brain structures are organized and quantify the brain’s ability to process information. In the global-level analysis of this study, both FC patients and HCs exhibited small-world properties (σ = γ/λ > 1, γ > 1 and λ ≈ 1). In a complex network, the small-world property is one of the topological characteristics of the brain structure network (17, 18), which has a high clustering coefficient and a long shortest path at the same time, which means that information transmission is efficient at the global and local levels (19, 20). This is similar to the results of previous studies. Both functional and structural networks of FC patients exhibit small-world network properties (15, 21). And, some scholars have shown that the γ and σ values of FC patients are significantly lower than those of HC in the functional network (21). However, this study’s results showed no statistically significant difference in information transfer ability between the two groups at the global level. This may be due to the effect of age on brain structure, that is, the white matter structure and network connectivity of the human brain decrease with age (22). The patient groups involved in previous studies were all younger, and the patients in this experimental group were older. It can be speculated that the global properties of the white matter network in middle-aged and elderly FC patients were not reduced compared with middle-aged and elderly HC patients due to superimposed age factors. Second, this may be due to the mutual compensation of information flows at the global level to maximize the efficiency of information processing in FC patients (15).

### 4.2 Local topological properties of FC patients

Changes in nodal properties of limbic system: The limbic lobe refers to the ring structure composed of the cingulate gyrus, hippocampal gyrus, and uncinate gyrus located on the inner side of the cerebral hemisphere. The limbic system mainly includes the temporal pole, the orbital surface of the frontal lobe, the anterior part of the insula, and the subcortical amygdala and limbic lobe, with extensive and close structural and functional connections between them. The limbic system is mainly related to the higher cortex and autonomic nervous system. When it is damaged, there may be dysfunction related to nerve, mental and visceral activities, such as slow response, abnormal emotion and memory, hunger and thirst, etc. At present, many scholars have used neuroimaging techniques to confirm the abnormalities of brain structure and function of the limbic system in FC patients. Hu et al. found that the cortical thickness of limbic system brain regions such as ventral and dorsal prefrontal gyri, frontal orbital gyrus, and cingulate gyrus in FC patients was significantly lower than that in healthy controls (13). Other scholars used resting-state fMRI combined with local consistency and low-frequency amplitude analysis to find that the brain functional activity of limbic system regions such as the parahippocampus and frontal orbital gyrus in FC patients was higher than that in healthy controls (23, 24). In addition, Liu et al. used fMRI combined with graph theory analysis to find that compared with HC patients, FC patients had significantly lower nodal degrees and nodal efficiency in the thalamus, anterior cingulate gyrus, and SMA brain regions, and the thalamus-related network and limbic system (amygdala and hippocampus) are also abnormal (14).

In this study, changes were found in the nodal properties of the frontal orbital surface, cingulate gyrus, and hippocampus in FC patients. The orbital surface of the frontal cortex is considered a neural bridge connecting the external and internal environments, which integrates the process of visceral sensory information (25, 26). As a key area involved in the conduction and integration of sensory information, the orbital surface of the frontal cortex receives multiple sensory inputs (gustatory, olfactory, somatosensory, auditory, and visual) and visceral sensory information (27), and is considered to play an important role in the integration of visceral sensorimotor information (28, 29). Functional and structural abnormalities of the frontal-orbital gyrus in patients with FC have been found in several neuroimaging studies (12, 13). For example, a recent structural MRI study showed that the frontal-orbital gyri of FC patients exhibited abnormal cortical thickness compared with HC (13), whereas fMRI studies found the altered activity of the same structures (23). The cingulate gyrus serves as an intermediate bridge to project fibers from the frontal orbital gyrus and insula to the SMA, amygdala, etc., and its front and ventral emotional functional areas can communicate with the internal organs and endocrine systems regulated by sympathetic and parasympathetic nerves, thus regulating gastrointestinal function and activities. In addition, the anterior cingulate usually responds to emotion and motivation by producing pain sensations and can respond emotionally to noxious stimuli (30, 31). The hippocampus is associated with higher brain activities such as remembering emotions (32). The amygdala is an important structure involved in checking, integrating, and filtering emotional information (33, 34). Abnormal changes in nodal properties of the limbic system in patients with FC were found: left Hippocampus (HIP.L), left amygdala (AMYG.L), left anterior cingulate and nodal cluster coefficient increased; right orbital middle frontal gyrus (ORBmid.R) nodal efficiency increased. These results suggest that there may be abnormal visceral sensorimotor and emotional processing in FC patients, and the significantly increased information transmission of the visceral motor network may cause constipation, resulting in negative emotions, and negative emotions become a harmful stimulus that reverses the gastrointestinal tract, leading to Constipation or worsening symptoms of constipation (35). For example, frequent defecation failure, abdominal discomfort and other adverse symptoms and increased defecation expectations may prompt FC patients to pay too much attention to the disease, resulting in abnormal sensory and emotional processing, which is usually shown as abnormal activity of information transmission in related brain regions, in order to avoid this vicious cycle, the brain area were negatively regulated (right dorsolateral superior frontal gyrus (SFGdor.R), right orbital inferior frontal gyrus (ORBinf.R), right medial superior frontal gyrus in FC patients (SFGmed.R), right medial and paracingulate gyrus (DCG.R), right hippocampus (HIP.R), left paracentral lobule (PCL.L), and left temporal pole (TPOsup.L) nodal Clustering coefficient and nodal local efficiency decrease) to maintain the balance of visceral movement and emotional response. In addition, in the FC patient group, the nodal efficiency of ORBmid.R was positively correlated with the PAC-QoL score (*r* = 0.55, *p* = 0.002, which can be corrected by FDR), and the PAC-QoL score scale can well evaluate the Quality of life and disease severity, the correlation between the two shows that the quality of life of FC patients is related to the increased information transmission efficiency of ORBmid.R and other nodes in the white matter network, suggesting that the enhanced ability of ORBmid.R to integrate visceral information is associated with disease severity in elderly FC patients. This is consistent with the concept of neuronal plasticity, that is, neurons change their structure and function in order to adapt to the influence of environment and experience on the nervous system; that is to say, the influence of long-term constipation on the human itself leads to the change of brain white matter structure, which affects the change of brain function (36).

Changes in nodal properties of supplementary motor brain areas: SMA is one of the important brain areas in the primate cerebral cortex, which not only regulates motor functions but also is sensitive to visceral sensory stimuli (37, 38). FGID-related studies have shown that compared with HC patients, FGID patients have increased functional connectivity between SMA and other brain regions. These results suggest that abnormal functional connectivity in SMA brain regions is associated with the preparation, and planning of motor response to the visceral anomalous stimulus (39–41). In addition, some scholars have confirmed through imaging studies that the voluntary contraction of the pelvic floor muscles can cause changes in the activity level of the SMA brain area (42, 43), and the pelvic floor muscles are one of the important muscles involved in the defecation process. When constipation occurs, the contraction of pelvic floor muscles increased, and the information transmission speed and functional activity of SMA also increased accordingly (the nodal local efficiency of left supplementary motor cortex (SMA.L) increased).

Changes in nodal properties of the default mode network: the default mode network (Default mode network, DMN) refers to certain brain regions that are activated in the human brain in a waking resting state (when not receiving external stimuli or performing tasks), these brain regions are extensively interconnected. The angular gyrus, precuneus, and cuneiform are the core brain regions of the default network. Some scholars have used structural MRI (20) to find that compared with healthy people, FC patients have decreased cortical volume involving the middle temporal gyrus (Middle Temporal Gyrus, MTG), precentral gyrus, and precuneus (precuneus, cuneus) in the DMN are, suggesting abnormal changes in the structure of the DMN in patients with FC. In addition, some scholars (44) used acupuncture to confirm the important role of the precuneus and angular gyrus in regulating the attention-inhibition network. By weakening the functional activities of the above two brain regions, they can control emotional ups and downs, thereby improving constipation. In this study, the decreased nodal properties in the right precuneus PCUN.R, left cuneus CUN. L, left angular gyrus ANG.R, and left parietal-inferior marginal angular gyrus IPL.R may be associated with depression in patients with constipation. The temporal lobe is another core brain area of the DMN, which is involved in the integration of semantic understanding and multimodal perception (24). Zhou et al. found that the signals of the temporal lobe cortex had changed through acupuncture experiments, suggesting that the abnormal changes of the temporal lobe may be related to the improvement of patients’ adverse symptoms and the reduction of excessive attention to the body (45). This may explain the fact that the increased node properties of the left transverse temporal gyrus (HES.L) in this study may be related to the increased concern and self-suggestion caused by constipation.

In summary, this study used DTI combined with graph theory to confirm the significant differences firstly in the topological properties of the limbic system, supplementary motor cortex, and default mode network brain regions in middle-aged and elderly FC patients, and revealed the changes in the white matter network of middle-aged and elderly FC patients. It also shows the abnormal and compensatory regulation of visceral sensorimotor processing and emotional processing in the central nervous system. Although the occurrence of a causal relationship between brain structural abnormalities in the white matter network and quality of life is unknown, the results provide for the first time visual evidence of brain structural alterations in middle-aged and elderly FC patients, deepening our understanding of the neuropathology of FGID.

## 5 Limitations

This study has certain limitations. First of all, the sample size of this study is small and no 3.0 T magnetic resonance scanner was used for DTI scanning, which affects the stability and accuracy of the research results. Secondly, this study is a study of white matter, and FC patients also have changes in gray matter structure and brain function, so multimodal comprehensive research should be carried out in the future.

## Data availability statement

The raw data supporting the conclusions of this article will be made available by the authors, without undue reservation.

## Ethics statement

The studies involving humans were approved by the Ethics Committee of Affiliated Xinhua Hospital of Dalian University. The studies were conducted in accordance with the local legislation and institutional requirements. The participants provided their written informed consent to participate in this study.

## Author contributions

HX: Data curation, Software, Writing – original draft. AQ: Data curation, Software, Writing – original draft. SC: Data curation, Writing – original draft. ZY: Data curation, Writing – original draft. WW: Conceptualization, Funding acquisition, Writing – review & editing.
